# Sectarianism at London Hospitals

**Published:** 1888-10-20

**Authors:** 


### The Hospital

A Weekly Institutional Journal of

**Science, Medicine, Nursing, and Philanthropy.**

*For Hospitals, Asylums, Medical Practitioners, Students, Nurses, Families, Parishes, Congregations, and the Charitable Public.*

Vol. V. No. 108.] [October 20, 1888.

### SECTARIANISM AT LONDON HOSPITALS.

A good deal of excitement has recently prevailed in certain quarters about a nurse who was said to have been refused employment at St. Thomas's Hospital on the ground of her religion. The nurse was a Roman Catholic. Some time ago a similar fire of religious zeal was kindled in connexion with University College Hospital. The nurse in that case was also a Nonconformist. The subject is one of wide bearings. It concerns on the one hand both the external support and the internal administration of hospitals, and on the other the broader questions of religious tolerance and national unity of sentiment.

No man who has made it a part of his duty to observe, and, if possible, understand his fellow-men, will deny the existence and the all-pervading pressure of sectarian energy. Men and women who are honestly and deeply religious are accustomed to regard religion, not as an accidental or secondary circumstance of life, but as the most fundamental and primary of all the conditions of healthy and right-minded humanity. The highest authority known to Christians distinctly affirmed this position. "What shall it profit a man," said Jesus of Nazareth, "if he gain the whole world and lose his own soul?" The sincere religionist sees the fundamental nature of the proposition, and very generally interprets it by his own ecclesiastical standpoint. What shall it profit a man, thinks he, if he gain the whole world but is not a Roman Catholic? What shall it profit him if he be not a Churchman, or a Congregationalist, or a Methodist, or a Plymouth Brother? The more intelligent few in each of these religious organisations recognise that the divine religion is broader than any or all of them; broad, indeed, as humanity. Whilst therefore they agree with the philosophic soundness of the New Testament position, they interpret it without sectarian limitations. But religious organisations, like towns and countries, consist of a strictly limited number who may be properly called thinkers, and these are associated with the indefinite crowds who take their opinions at second hand, without knowing or caring what their particular value may be. Just as a man whose pockets are filled with coppers makes a loud rattling as he runs along the streets, whilst he whose money consists in a large balance at the bank may make no noise at all; so does the religious man of secondhand opinions become the fiery zealot who persecutes and anathematises his neighbour, whilst his more thoughtful co-religionist deplores his intellectual incapacity and his want of moral self-restraint.

Sectarianism we regard as a fire which is never quite quenched but always smouldering, and only awaiting the circumstance of a favourable breeze to be fanned into a flame. Experience seems to warrant the conclusion, not only that it never has been quenched, but that it never will be. It is therefore to be regarded as a fact of human life which must always and everywhere be looked for and reckoned with. It is, moreover, almost always a dangerous circumstance. The born sectary is generally a fanatic, and the fanatic has usually thrown overboard his reason on religious questions; and, what is more, he boasts of the fact. The only thing, therefore, that practical and soberminded men can do, if they desire the world's business to be conducted fairly and without friction, is to be always on the watch for Sectarianism, and always prepared to hold it as much as possible in check.

This is affirmed as a rational working position, which most reasonable Englishmen, who are by nature as tolerant as they are phlegmatic, will decidedly endorse. How do the London hospitals appear when tried by such a standard? It so happens that facts are available which enable a definite and convincing answer to be given to the question. Not more than two years ago the action of University College Hospital, already alluded to, caused an amount of excitement infinitely greater than that recently aroused at St. Thomas's. The Hospitals Association then took up the subject, and instituted a thorough investigation. A carefully-prepared series of printed questions was sent to every hospital in and around London. The questions were so framed that the hospital authorities were compelled either to disclose the real facts connected with the public and private religious usages of their institution, or to decline giving any information at all. As a matter of fact, however, not only did the hospitals as a whole furnish the fullest possible information, but, with one or two exceptions, they evinced the utmost anxiety to clear themselves of the least suspicion of Sectarianism. The voluntary hospitals, it may be said, had good reasons for doing this, which could not apply to the endowed charities, such as St. Bartholomew's, St. Thomas's, and Guy's. But the latter showed themselves quite as eager to disclaim Sectarianism as the former; more anxious, indeed, than one or two of the voluntary hospitals. The particular member of The Hospitals Association who conducted the investigation had a peculiar interest in discovering the real truth, inasmuch as he himself belonged to the same religious organisation as the young woman who had been so cavalierly treated at University College. He, as well as other members of the Association, came to this general conclusion, that, on the whole, the London hospitals were not sectarian institutions; that, in fact, the authorities had a very strong desire and determination to discountenance and discourage Sectarianism absolutely. There might be among the hundreds of matrons, sisters, and other officials some few individuals of strong ecclesiastical proclivities who occasionally caused a little inconvenience; but that is inevitable everywhere and under all circumstances. What was conclusively proved was that the laws and usages of London hospitals were such as to do substantial justice to nurses and patients of all sects and parties. Whatever may have been the actual circumstances which occurred between the matron and the Roman Catholic nurse at St. Thomas's we do not know, for only one side of the case has been heard. This, however, we do know, and that on the best authority, that the governors of St. Thomas's have no desire whatever to interfere with anybody's religious convictions; and that their regulations are framed on principles of the broadest toleration.

There is a disposition on the part of some hospital managers to make light of the religious difficulty, and to look upon people who have strict religious convictions rather as troublesome nuisances. It must be admitted that an energetic denominationalist, whether she is a matron or a nurse, is often decidedly in the way. She has the inconvenient faculty of obtruding her peculiar views at all sorts of appropriate and inappropriate times. She does not understand the principle of live and let live. It is a fact that many nurses who make no particular profession of religion at all are much more serviceable in their profession and much less difficult to manage than some who make great show of devoutness. By no means is all the intolerance found on the side of matrons and officials. Nurses themselves can and do sometimes make a very great noise about a very little circumstance. We do not counsel irreligion, or think that, as a rule, irreligious persons do their work better than those who are religious. But there are such things as good sense, patience, meekness, and good will, which go a long way towards smoothing over difficulties of all kinds, religious and otherwise. It is a matter well worthy of consideration on the part of nurses how much of their own distinctive peculiarities they can sink without injury to conscience and self-respect.

But, on the other hand, those hospital authorities, if there are any such, who regard with indifference, not unmixed with contempt, the distinctive religious convictions which may govern the conduct of some of their nurses, show a great want of intelligence, and a decided unfitness for their peculiar duties. If it be proper to call upon nurses, all of whom are women, and many of whom are but poorly educated, to show a reasonable and intelligent patience and modesty under circumstances of difficulty and opposition, much more is it right and imperative that governors, committee men, and the higher classes of officials shall evince a disposition and a capacity for statesmanlike breadth and practical administrative skill. Sectarianism and religious intolerance are always and everywhere both stupid and hateful to the intelligent mind. Nothing is more sure than this, that no man or woman can ever be the complete measure of any scientific truth; much less then can he be the complete measure of every religious truth. The only way in which men can live together in general society is by the exercise of mutual respect. How infinitely more essential is the mutual respect when men and women have to live together daily within the same four walls, and to work together daily at the bedside of the sick, the sorrowful, and the dying. Hospitals, of all places, should see no part of religion except those practical aspects which reveal its purity, its simplicity, its helpfulness, and its love.

